# Traumatic testicular injuries in appalachia: A ten-year review from a level 1 trauma center and comparison to the national trauma data bank^®^

**DOI:** 10.3389/fruro.2023.1105513

**Published:** 2023-01-24

**Authors:** Katharina Mitchell, Chad Morley, John Barnard

**Affiliations:** West Virginia University Department of Urology, 1 Medical Center Dr, Morgantown, WV, United States

**Keywords:** trauma, testicular, rural, orchiectomy, injury

## Abstract

While only 20% of the nation’s population lives in rural areas, 40% of the Appalachian population resides in rural areas. Due to the rural nature of this region we hypothesized this may have implications regarding the outcomes of traumatic testicular injuries (TTI), such as increased rate of organ loss. Here in, we report the findings of our 10-year retrospective study analyzing patients presenting with TTI to our rural tertiary care facility in comparison to an 8-year review of 8,030 TTI from the National Trauma Data Bank (NTDB^®^). Of the 34,000 trauma patients reviewed, 23 (0.07%) had TTI which concurs with the NTDB^®^ value of 0.2%. Blunt trauma accounted for 91.3% of TTI contrasting with NTDB^®^ data suggesting 50.5% attributable to penetrating mechanisms. Firearm related injuries comprised 4.3% of TTI, but 38.3% of NTDB^®^’s. Motor vehicle collision/motor cycle crash (MVC/MCC) accounted for 26.0%, similarly NTDB^®^ data showed 26.6%. Median length of stay (LOS) was 1 day, and 3 for NTDB^®^. Scrotal exploration occurred in 90.4% of cases with 52.4% requiring orchiectomy, whereas NTDB^®^ data suggested 48.3% and 23.4% respectively. In conclusion, compared to the NTDB^®^ TTI data, Appalachia has a higher incidence of blunt mechanism, scrotal exploration rate, and testicular loss.

## Introduction

Male traumatic genital injuries are relatively uncommon and are typically not life-threatening when considered in isolation. Testicular injury has been shown to occur in less than 1% of trauma (1, 2). While not life-threatening, traumatic testicular injuries (TTI) often result in organ loss and morbidity associated with genital injury. The scrotum lies in a dependent nature which can make it susceptible to injury, however, its mobility is protective against severe injury (2). Trauma inflicted on the testicles can result in testicular torsion, intratesticular hematoma without rupture, testicular rupture/fracture, and testicular dislocation- all of which require emergent management apart from intratesticular hematoma (3). Degree of injury can be difficult to assess solely based on physical exam following scrotal trauma due to potential swelling, ecchymosis, lacerations and skin loss. Assessment of choice is ultrasound (U/S) which has a high sensitivity and specificity in detecting clinically significant testicular injury (2). U/S has a reported sensitivity of 50%-100% and specificity of 65%-89% depending on type of testicular trauma in question (4).

With regards to management, the American Urologic Association (AUA) 2017 guidelines state “surgeons should perform scrotal exploration and debridement with tunical closure (when possible) or orchiectomy (when non-salvageable) in patients with suspected testicular rupture” (5).

The present study highlights traumatic testicular injury (TTI) data from a rural tertiary care facility in Appalachia. The Appalachian region is defined as the 205,000-square-mile region that follows the spine of the Appalachian Mountains from southern New York to northern Mississippi. Appalachia encompasses parts of 12 different states including: Alabama, Georgia, Kentucky, Maryland, Mississippi, New York, North Carolina, Ohio, Pennsylvania, South Carolina, Tennessee, Virginia- and finally all of West Virginia. While only 20% of the nation’s population is rural, 40% of the Appalachian population is rural (6). With the TTI data from our rural tertiary care facility we will compare the patient population to the aggregate data presented by the National Trauma Databank^®^ (NTDB^®^). The purpose was to evaluate TTI at our rural tertiary care facility and compare the results to an 8-year review of 8,030 TTI from the National Trauma Data Bank (NTDB^®^) by Grigorian et al. (1). We hypothesized the data gathered from our institution may differ from that of the NTDB^®^ 2018 evaluation due to the disproportionately rural nature and prolonged transfer times.

## Materials and methods

In this retrospective review 34,000 male trauma patients that presented between January 2008- January 5^th^ 2019 were evaluated, and 23 (0.07%) of those patients had TTI. No age restrictions were made on inclusion criteria for eligibility to this study. Information was collected from the electronic medical record used at our rural tertiary care academic center whose 2 million patient catchment area lies entirely within the boundaries of Appalachia. The following parameters were included: patient age (years), injury date, ED date, ED arrival time, if the patient was presenting as a transfer, distance of transfer (miles), length of hospital stay (days), injury type (blunt vs penetrating), mechanism of injury, Injury Severity Score (ISS), abbreviated injury scale (AIS) head, AIS neck, AIS thoracic, AIS abdominal, AIS spine, AIS upper extremity, AIS lower extremities, AIS external, laterality and disposition from the ED. Parameters also included the presence of whether a focused assessment with sonography in trauma (FAST) was performed, if imaging was suggestive of injury at time of presentation, and concomitant genitourinary (GU) injury. Operative data including time to operative intervention and outcomes, if orchiectomy performed, if contralateral orchiopexy performed, transfusion requirement, follow up imaging, and urology follow up were also assessed. AIS scores range from 1 (minor) to 6 (maximal) for an individual injury to a body lesion and are an internationally accepted method to classify injury (7). Injury Severity Score (ISS) is based on the AIS, however, it examines the combined effects of patients with multiple injuries (8). Both ASS and IS, as described by NSW Institute of Trauma and Injury Management, were calculated using MDCalc. Data was averaged using Microsoft Excel.

## Results

Over 34,000 male trauma patients were reviewed, and 23 (0.07%) were found to have TTI. The age range of patient’s studied was 3-69. The mean age of men in our study was 29.4 years and median ISS was 4.0. Blunt trauma accounted for 91.3% of TTI. Firearm related injuries comprised 4.3% of TTI in the Appalachian cohort whereas MVC/MCC (26.0%), sports related (26.0%), work site (21.7%), and straddle (13.0%) mechanisms were far more prevalent. While not strictly seen as traumatic injury, testicular torsion or partial torsion was seen in 3 out of the 23 cases (13.04%) presented. While testicular torsion is typically seen in patients without traumatic history, the patients included in this database with torsion developed torsion secondary to trauma such as wrestling. Transfers accounted for 35% of patients with mean distance of 59.8 miles (**Table 1**). Of the 8 patients (35%) that were transfers, 87% went to OR and 50% of those who went to the OR underwent orchiectomy. Of the non-transfers 60% (9/15) went to OR with urology, 40% (7/15) unilateral complete orchiectomy, and 6.7% (1/15) partial orchiectomy. Median LOS was 1 day for the Appalachian cohort with 90.4% of patients who survived the primary trauma evaluation undergoing scrotal exploration and 52.4% requiring orchiectomy. Concomitant GU injury was seen in 3/21 (14%) Appalachian patients who survived the initial trauma evaluation. Data is summarized in **Table 2**.

**Table 1:** Demographic and clinical data averages of entire rural cohort.

Average Age	29.4
% of patients who presented same date as injury	19/23 (82.6)
Average delay of days between injury date and ED presentation date in those who delayed	2.5 days
Amount of TTI patients who were transfers	34.7%
Average transfer distance	59.8625 miles
Average duration from ED presentation to OR	5.89 hours

**Table 2:** Comparison of the Appalachian TTI cohort to eight year NTDB ^®^ Data by Grigorian et al.

Characteristics	NTDB^®^ (n=8,030)	Appalachian Cohort (n=23)
Total trauma patients n	3,489,850	34,000
Age (years, median)	31.0	20
Penetrating Mechanism n (%):	4,054 (50.5)	1 (4.3)*
- Firearm	3,073 (75.8)	1 (100)
Blunt Mechanism n (%):	3,579 (44.6)	21 (91.3)
- MCC/MVC/motorcycle	2,140 (59.8)	6/23 (26.1)
- Sport/bicycle/pedestrian	Bike=100 Pedestrian= 567 Total= 667 (18.6)	6/23 (26.1)
Genitourinary Injury n (%):		
- Isolated scrotal	5,986 (74.5)	NA
- Penile	1,300 (16.2)	3/21 (14.3)
- Bladder or urethra	750 (9.3)	3/21 (14.3)
- Kidney	222 (2.8)	1/21 (4.8)
- Ureter	53 (0.7)	1/21 (4.8)
Surgical Outcomes n (%):		
- Scrotal exploration	3,882 (48.3)	19/21** (90.4)
- Unilateral orchiectomy	- (23.4)	11/21 (52.4)
- Contralateral orchiopexy	- (-)	5/21 (23.8)
- Penile operation	716 (8.9)	0/21 (0)
LOS, days median	3	1
Mortality	609 (7.6)	NA

* 1 patient in our study was neither blunt nor penetrating (awoke with scrotal pain).

** 2 patients were not included for surgical considerations because they died from concurrent injuries.

## Discussion

When compared to the NTDB^®^ TTI data, Appalachia has a much higher incidence of blunt mechanism, accounting for 91.3% of TTI, while the prevalence was only 44.6% in the NTDB^®^ study (1). Our center’s referral base is the 29^th^ most densely populated state in the US and includes approximately 2 million people (9). Additionally, it lies entirely within Appalachia which suggests an accurate representation of the entire Appalachian region. The NTDB^®^ included a large range of trauma centers, many of which were in urban settings that receive many penetrating injuries due to higher interpersonal violence rates. Therefore, our data revealing significantly more blunt than penetrating trauma in comparison to that study could be due to the nature of our population. However, the findings of increased prevalence of blunt TTI mirrors multiple previous studies which attributed 63% to 85% of TTI to blunt trauma (2, 10). We found MCC/MVA and sports injuries were the most common culprits of blunt testicular trauma, each causing 26.1% of the total blunt injuries. The most common sports associated with injury were lacrosse (2/6) and wrestling (2/6). In a study by Altarac et al. it was found that 18% of athletes experienced a testicular injury during participation in sports. Testicular injuries were most common in lacrosse and wrestling with 48.5% and 32.8% of respondents indicating these as the sport in which injury occurred, respectively (11). Another difference discovered between our study and the NTDB^®^ was a higher scrotal exploration rate, and increased likelihood of testicular loss. The finding may be attributed to the rural nature of our state which consists of numerous small, critical access hospital and long transfer times prior to definitive care by the appropriate subspecialist. Most of West Virginia’s land mass is designated as rural, and overall the state’s population is designated as 51% rural and 49% urban (9). Testicular traumas including torsion, testicular fracture/rupture, and testicular dislocation are all time sensitive. In a study by Howe et al, looking at 81 males who presented with testicular torsion, only duration and age were correlated with the risk of non-salvage on multivariate analysis (7). It has been established that when surgical exploration is delayed, testicular atrophy will occur by 6 to 8 hours, and necrosis within 8 to 10 hours of initial presentation. When surgical exploration is undertaken within 6 hours of symptom onset salvage rates are over 90%. However this dramatically decreases to 50% when symptoms persist over 12 hours, and subsequently to less than 10%, when symptoms have been present for over 24 hours (12). Where testicular rupture is concerned, when surgery is done within the first 72 hours of symptom onset salvage rates are up to 90%, which decreases to approximately 45% when intervention is delayed over 72 hours (13). There were higher scrotal exploration and testicular loss rates in our study in comparison to the NTDB^®^ which confirmed our hypothesis that there would be a higher rate of testicular loss in our catchment. While the impact of rurality on testicular loss can not directly be deduced, further exploration of a possible correlation would be of value. Additional examination of the nature of testicular trauma in rural America might be better captured though collaborative data collection among the largest referral centers in rural areas.

## Contributions

This contributes to the literature as an illustration of a higher testicular loss and exploration in comparison to the NTDB as seen in this rural setting, which sparks subsequent need for additional exploration of possible contributing factors. Future interest to rural care providers may focus on the availability of u/s at smaller referral centers and distance traveled by patients prior to first evaluation in addition to distance and time in transport thereafter to health center with ability for definitive management of patient’s pathology.

## Data availability statement

The original contributions presented in the study are included in the article/supplementary material. Further inquiries can be directed to the corresponding author.

## Author contributions

KM- wrote manuscript and complied data. CM- edited manuscript. JB- edited manuscript, complied data, senior author. All authors contributed to the article and approved the submitted version.
